# Spatial and temporal dynamics of shifting cultivation in the middle-Amazonas river: Expansion and intensification

**DOI:** 10.1371/journal.pone.0181092

**Published:** 2017-07-20

**Authors:** Catarina Conte Jakovac, Loïc Paul Dutrieux, Latifah Siti, Marielos Peña-Claros, Frans Bongers

**Affiliations:** 1 Instituto Nacional de Pesquisas da Amazônia, Manaus, Brazil; 2 Forest Ecology and Forest Management Group, Wageningen University & Research, Wageningen, The Netherlands; 3 Laboratory of Geo-Information Science and Remote Sensing, Wageningen University & Research, Wageningen, The Netherlands; Chinese Academy of Forestry, CHINA

## Abstract

Shifting cultivation is the main land-use system transforming landscapes in riverine Amazonia. Increased concentration of the human population around villages and increasing market integration during the last decades may be causing agricultural intensification. Studies have shown that agricultural intensification, i.e. higher number of swidden-fallow cycles and shorter fallow periods, reduces crop productivity of swiddens and the regrowth capacity of fallows, undermining the resilience of the shifting cultivation system as a whole. We investigated the temporal and spatial dynamics of shifting cultivation in Brazilian Amazonia to test the hypotheses that (i) agriculture has become more intensive over time, and (ii) patterns of land-use intensity are related to land accessibility and human population density. We applied a breakpoint-detection algorithm to Landsat time-series spanning three decades (1984–2015) and retrieved the temporal dynamics of shifting cultivation fields, which go through alternating phases of crop production (swidden) and secondary forest regrowth (fallow). We found that fallow-period length has decreased from 6.4 to 5.1 years on average, and that expansion over old-growth forest has slowed down over time. Shorter fallow periods and higher frequency of slash and burn cycles are practiced closer to residences and around larger villages. Our results indicate that shifting cultivation in riverine Amazonia has gone through a process of agricultural intensification in the past three decades. The resulting landscape is predominantly covered by young secondary forests (≤ 12 yrs old), and 20% of it have gone through intensive use. Reversing this trend and avoiding the negative consequences of agricultural intensification requires land use planning that accounts for the constraints of land use in riverine areas.

## Introduction

Tropical landscapes have been largely transformed by human land use. Shifting cultivation plays a central role in land cover transformation given its widespread occurrence in the tropics [1]. Such landscapes are characterized by a mosaic of swiddens (temporary cropping fields) and fallows (temporary secondary forests) that are dynamic through space and time, and are mainly managed by slash and burn practices. Across the tropics, shifting cultivation systems have undergone expansion and intensification phases following public policies and socio-economic changes. From the 1960s to the 1980s, shifting cultivation was expanding over forests following government policies that stimulated the colonization of agricultural frontiers in Asia and Latin America [2]. Subsequent public policies discouraged shifting cultivation practices and triggered, together with population increase and other socio-economic drivers, agricultural intensification and the replacement of swiddens for permanent land uses [3, 4].

Processes of expansion and intensification in shifting cultivation have been widely reported for Asia (for a review see [5]), but less so for South America and especially for Amazonia [5–7]. The overall low population densities in Amazonia have raised little concern and scientific attention to land use and land cover changes related to shifting cultivation, which remains the main agricultural system in riverine areas [5]. Despite the apparent large availability of land in riverine Amazonia, access restrictions may play an important role in ruling actual land availability and resource use in these areas where land is accessed by walking and by permanent and intermittent rivers [8–10]. Additionally, in the last decades riverine Amazonia is experiencing important changes in its socio-economy and demography [7]. In search for better life conditions, people are migrating from the headwaters to closer to the urban centres, resulting in a rapid urbanization and increased demographic concentration around villages and around small urban centres [11–13]. Coupled with the urbanization process, market demand for cassava flour (*farinha*, in Portuguese), the staple food in the Brazilian Amazonia, is increasing and is leading to changes in traditional agricultural practices [14, 15]. Since access, migratory movements, market demand and price changes have been historically important determinants of social changes with strong implications for land use and land cover [7, 16], it is expected that these forces are also affecting land use patterns and encouraging agricultural intensification in riverine Amazonia.

In this study, we investigate the temporal and spatial dynamics of shifting cultivation in the region of the middle-Amazon river to evaluate how shifting agriculture has expanded and/or got intensified in the last three decades. The middle-Amazon river is one of the largest cassava producing region in the Brazilian Amazonia, and is experiencing strong market orientation following the increasing market demand for *farinha* [17]. We hypothesized that shifting cultivation in the middle-Amazon river has become more intensified in the last three decades and that land accessibility and human population size play important roles in determining land use and land cover patterns.

We used the product of a novel remote sensing approach based on time-series analysis to detect land cover changes and retrieve the frequency of slash-and-burn events and the length of fallow periods over time (important indicators of agricultural intensification [18]). Most remote sensing studies rely on land cover classification and bi-temporal changes, i.e. compare the land cover in two moments in time (e.g. [19]). While these methods are useful for detecting major changes in deforestation and the spatial distribution of different land cover classes, they are limited in recognizing changes in highly dynamic systems in the tropics, where rapid vegetation regrowth limits their ability to distinguish between land cover types such as cropping fields and secondary forests [19, 20]. To overcome this limitation, we applied the B-FAST algorithm that allows for constructing time-series using every satellite image available for a given area and for segmenting this time-series into homogeneous temporal trajectories, based on the detection of breakpoints that represent changes in land cover [21, 22]. Variations of this method have been recently applied to monitor disturbance in old-growth forests [23, 24], and are considered promising to overcome the challenge of detecting land cover changes in highly dynamic systems in the tropics [20, 25, 26].

From the remote sensing time-series we retrieved and mapped the history of clear-cut events in swidden-fallow fields during the last three decades (1984 to 2015) to evaluate (i) how fallow period and (ii) expansion over old-growth forest changed over time, and (iii) how land use patterns are related to land accessibility and human population density. Additionally, we describe the current shifting cultivation landscapes in terms of land-use intensity and age distribution of secondary-forest-fallows. Understanding how shifting cultivation is being transformed in riverine Amazonia is essential to inform land-use planning and agriculture policies and to improve estimations of the potential of these landscapes to provide ecosystem services, such as agricultural production and biodiversity conservation.

## Material and methods

### Study site

This study focused on the region of the middle-Amazon river, in the municipalities of Alvarães and Tefé. Tefé is the main urban centre in this region, with a population size of 62,662 inhabitants and density of 2.6 inhabitants per km^2^ [27]. It is the second cassava-flour producer in the Amazonas State, producing ca. 96,000 ton per year [27]. *Farinha* is the staple food in Brazilian Amazonia. It is produced from bitter cassava cultivated on swiddens in shifting cultivation systems by *caboclos*, who are descendants from the mix between indigenous and non-indigenous people [28]. Swidden cultivation in the study area is supported by family labour, with no use of external inputs such as fertilizers and herbicides, and is meant for subsistence and commercialization [15].

Riverine villages along the Tefé river have between one and 40 households [13]. After cassava flour, Brazil nuts and government subsidies are important sources of income [17, 29]. Although livelihoods are mainly supported by agriculture, fishing and hunting, there is also a high dependency on industrialized products, fuel, and tools. For this reason, visits to the urban centre of Tefé are becoming more and more frequent, following a trend of multi-sited households in riverine Amazonia [11].

Shifting cultivation landscapes located within 50 km of the urban centre of Tefé, and which are exclusively accessed by rivers and not connected by roads to the urban zone, are the focus of this study. We have chosen to study riverine communities located close to the urban centre because those are suffering faster transformation over time than the ones further upriver [13, 29].

The Brazilian Nacional Committee in Ethics in Research (CONEP; Br) and the Ethics Committee of the National Institute of Amazonian Research (Comissão de Ética em Pesquisa do Instituto Nacional de Pesquisas da Amazônia, CEP/INPA; Br) have approved this study and the assessment of land-use history by farmers’ interviews (project number 20361713.7.0000.0006). All interviewed farmers provided their written consent, which had been previously approved by CONEP and CEP/INPA.

### Detecting and mapping land-use trajectories

To detect land cover and land use changes over time, we used the product of a remote sensing study that reconstructed the full land-use history of this region [30]. The land-use history was retrieved from time-series using 350 images collected by the Landsat sensors Thematic Mapper (TM), Enhanced Thematic Mapper Plus (ETM+) and Operational Land Imager (OLI), spanning the 1984–2015 period and with a 30 m ground resolution. The study area was assessed in the Landsat scene path 1 row 62. We used all images available for this scene over time to construct a detailed time-series. For images partially covered with clouds we have excluded the pixels contaminated by clouds using the *fmask* algorithm but kept the cloud-free pixels (see [30]). We have also masked out the area covered by old-growth forest in the most recent satellite image (August-2015), to build time-series only on areas that had been through some type of land-use change.

We used the Breaks For Additive Season and Trend (BFAST) framework for automatically detecting abrupt land-cover changes in remote sensing time-series (Verbesselt 2010). We combined the BFAST approach with a multi-temporal spatial segmentation, to provide information at the object level (i.e. at the scale of individual swidden-fallow fields) [30]. We characterized land cover with the Normalized Difference Moisture Index (NDMI) because it is appropriate for detecting regrowth [30]. The main steps of the method consisted in (i) assembling multiple layers of NDMI derived from Landsat data into time-series, (ii) segmenting the area by grouping neighbouring pixels with similar land-use trajectories, (iii) detecting break points in the NDMI time-series for each segment independently, using the BFAST algorithm, and (iv) classifying the detected breaks according to the change-process they represent using a Random Forest Classifier. The detected breaks represent changes in land use regimes, such as clear-cuts (i.e. slash-and-burn event) or land abandonment/stabilisation (S1 Fig).

The Random Forest Classifier [31] was trained based on information acquired from ground truth and interviews with farmers on the land-use history of the fields (for details see [18]). In this study we are interested in the breaks representing clear-cut events because they represent the start of a new swidden-fallow cycle. After identifying the dates of clear-cut breaks for each segment (called *field* from now on) we then retrieved (i) the number of swidden-fallow cycles (i.e. number of times the field had been cultivated), and (ii) the duration of the fallow period between two clear-cut events, throughout the monitoring period. The applied method predicted the number of swidden-fallow cycles with high accuracy (NRMSE = 0.25), and the length of the fallow period with an accuracy of 1.3 years (NRMSE = 0.19) [30, 32]. For detailed explanation on the remote sensing procedures for detecting land-cover trajectories, and for performing the spatial and temporal segmentation see [30].

Because the BFAST approach does not identify the land cover at the start of the monitoring period, we have classified a 1984 reference image (Landsat image from August 9^th^, 1984) into six land cover classes: old-growth forest, agricultural field (including fallows and cropping fields), bare soil, cloud, shadow and water. We used a Random Forest Classifier [31] trained with a visually interpreted dataset from the same image. We used an Out of the Bag validation which indicated a high accuracy (0.44% estimated error) and a high spectral separability of the land cover classes (estimated error between classes varied from zero to 0.08). We used this single-image classification to learn what the land cover was at the beginning of the time-series and to verify patterns of expansion of swidden-fallow fields over old-growth forest through time.

### Data analyses

#### Characterizing the active shifting cultivation landscape

**Fig 1:**
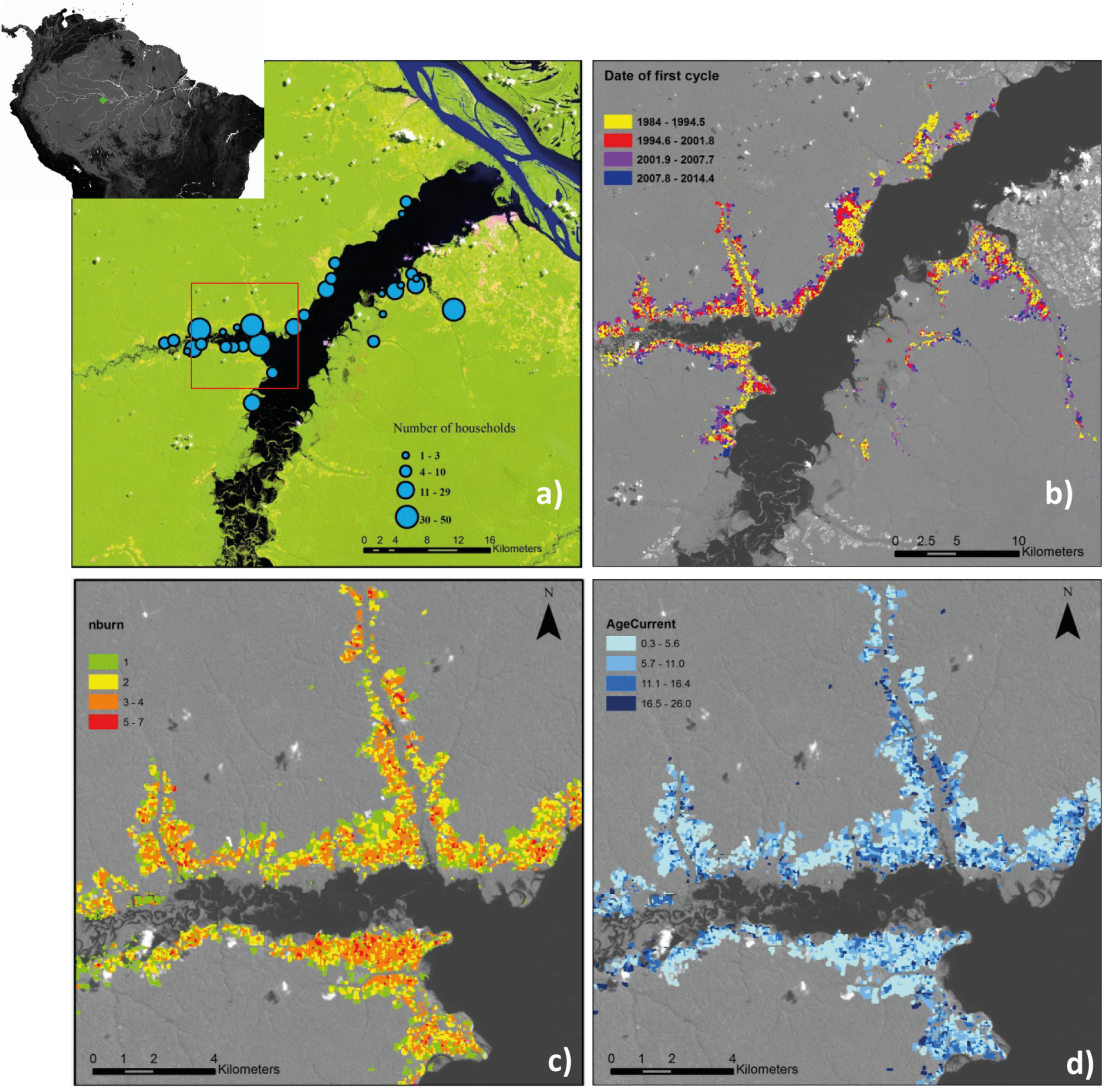
Map of the study area and the land-use history of the active shifting cultivation landscape retrieved by remote sensing. (a) Map of the Amazon Basin indicating the location of the study area (green diamond). Landsat 8 image (September 29^th^ 2014; band combination 6/5/2) of the study area (buffer zone) showing the location of riverine communities and respective number of households. The red square indicates the zoomed area represented in maps c and d. Maps show the land-use history of agricultural fields during the time period of 1985 until 2015. Segments represent pixels that have similar land-use history; (b) Map of the study area showing all the segments belonging to the swidden-fallow fields, i.e. which had at least one clear-cut event detected over the 1984–2015 period. Segments are coloured according to the date of the first clear-cut event detected by the BFAST analysis; (c) Map of the number of clear-cut events (slash and burn events) detected at each segment during the monitoring period; (d) Map of the age of secondary-forest-fallows in 2014 for each segment.

Using the time-series and segmentation approaches we segmented the study area into 13,840 fields (10,520 ha), from which 10,863 fields (8,259 ha) have had at least one clear-cut break detected over the 30 years period, and therefore are part of the active shifting cultivation landscape and are the focus of this study (Fig 1). The B-FAST approach requires a minimum number of observations for a breakpoint to be detected across the time-series profiles. For this reason, clear-cut breaks could only be detected between July 1987 and August 2014, so the actual monitoring period spanned 27 years.

For each swidden-fallow field within the active shifting cultivation landscape, we measured the area (in hectares), extracted all dates of clear-cut breaks detected over the monitored period (1987–2014), and assessed its land-cover class at the start of the monitoring period (1984). Based on these information, we assessed (i) the total number of swidden-fallow cycles at each field by counting the number of clear-cut breaks detected between 1987 and 2014, (ii) the length of the fallow periods, which were calculated as the time interval between subsequent clear-cut breaks subtracting the two years corresponding to the usual cropping period practiced in the study area [15], (iii) the age of the secondary-forest fallow in August 2014 (current secondary-forest age), (iv) the amount of area cultivated per year, calculated as the sum of the area of fields that experienced a clear-cut break in each year, and (v) the annual deforestation, calculated as the area of fields opened in old-growth forest, i.e. fields that experienced the first clear-cut break in that year and had been classified as old-growth forest in 1984. We describe the expansion of swidden-fallow fields over old-growth forests (deforestation) by the total area deforested in each year, the total area cultivated in each year, and the percentage of the cultivated area that was deforested in each year. We apply linear correlations to describe trends in the amount of cultivated area and annual deforestation over time.

#### Sampling within the landscape

We took a random sample of the active shifting cultivation landscape to be able to apply statistical analyses to evaluate determinants of land use pattern and changes in fallow period over time. We sampled fields within the landscape by randomly placing 2,451 points, using ArcGIS 10.1 [33]. This number of samples was the maximum number of points fitting the active shifting cultivation landscape given the following rules. To avoid sampling the same field more than once, we allowed a minimum distance of 70 m between points and have eliminated multiple points located inside the same field. For each sampled field we also measured its area in hectares and measured the Euclidean distance to the centre of the housing area of the nearest community, here forth named distance to residences, using ArcGIS 10.1 [33]. We assessed the location of riverine communities and its number of households during field expeditions in 2012 and 2013 [18] and complemented with secondary data [13].

#### Determinants of land-use patterns

We fitted mixed-effects models to evaluate how land accessibility and human-population size were related to the current age of secondary-forest fallows and to the number of past swidden-fallow cycles. We included as fixed factors the distance to residences (continuous variable, in km) and the community size (categorical variable), and communities as random factor (31 communities). Communities were classified according to the number of households it contains: isolated households (1–3 houses), small village (4–10 households), medium size village (11–29 households), and large village (> 30 households). Selection of random structure and the best mixed-effects models were based on the lowest Akaike information criterion (AIC) estimated using maximum likelihood (ML) method and on the most parsimonious model. The final best model was re-fitted using restricted maximum likelihood method (REML) for unbiased parameters estimates [34]. Mixed models were performed using nlme and MuMIn packages for R [35].

#### Changes in fallow-period length over time

To evaluate if the length of the fallow period has changed over time, we applied a survival analysis [36]. Such analysis has been applied before for this purpose elsewhere [8, 37]. This analysis estimates the survival probability of subjects that have been monitored through time and which could have experienced none, one or multiple events during the monitoring period. In this study, the event of interest is the clear-cut break detected by the BFAST approach, which represents the end of the fallow and the start of the next swidden period. The probability of survival refers to the probability of a field to continue as forest (old-growth or fallow), i.e. the probability of not been cut. In this analysis, the time period between the last clear-cut event and the end of the monitoring period is accounted as censored data [36].

We divided the monitoring period in two: 1984–2000 and 2001–2015, and fitted Kaplan-Meier survival curves for each time-period. We applied a log-rank test to evaluate if the probability of a fallow not been cut differs between time-periods. We fitted survival curves with the function *survfit* and tested log-rank differences with *survdiff* from the *survival* package for R [35].

## Results

### The active shifting cultivation landscape

The active shifting cultivation landscape totalizes 8,259 ha surrounding 31 riverine communities. Fields are located along the large rivers and small tributaries (Fig 1A, Fig 1B), with 80% of the fields comprised within 2 km from settlements (2.06 ± 1.65 km; Mean ± SD) (S2 Fig), mostly accessed by foot, while further away areas are accessed through small river tributaries (Fig 1C, Fig 1D).

**Fig 2:**
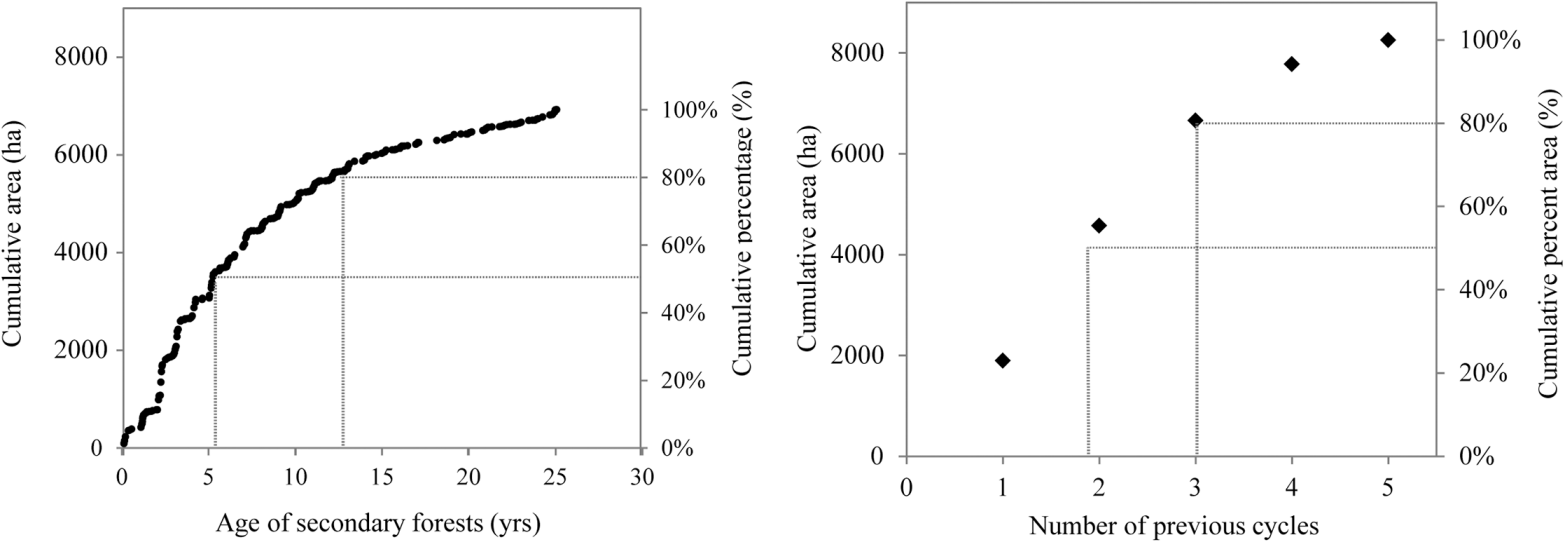
Secondary forest age and land-use intensity of the shifting cultivation landscape in 2014. (a) Cumulative area with secondary-forest fallows with different ages in 2014. (b) Cumulative area of fields that experienced different number of clear-cut events (swidden-fallow cycles). Dashed lines indicate the age of secondary forests (a) and the number of clearcut events in 50% and 80% of the fields.

In 2014, the active shifting cultivation landscape was mainly covered by secondary forests (84%; 6,929.34 ha), with only 16% of the fields being at the cropping phase (1,329.94 ha). Considering the current management in the study site [15], we classified as being at the cropping phase all fields within two years after the clearcut. Most of the secondary-forest fallows in the landscape (80%) were ≤ 12 years old (Fig 2A). The swidden-fallow fields have been cut, burned and cultivated a different number of times during the monitored period: 81% of the fields (6,659.8 ha) had experienced ≤ 3 clear-cut events within the monitored period (Fig 2B).

### Expansion of swidden-fallow fields over time

From 1987 till 2014 a total of 6,023 ha of old-growth forest had been deforested for shifting cultivation in the study area. In the reference image of 1984, most of the *active shifting cultivation landscape* was covered by old-growth forest (73%; 6,023.2 ha), 24% (1,958.9 ha) was already deforested, and the remaining area could not be classified due to cloud coverage (3%; 277.2 ha).

**Fig 3:**
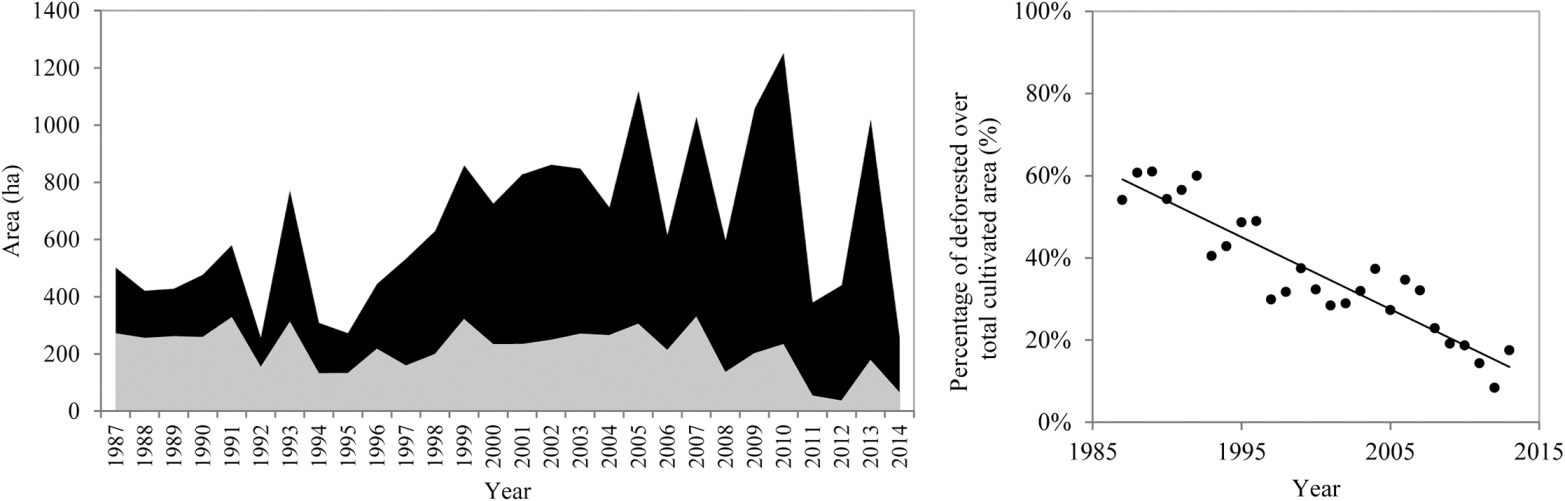
Expansion of shifting cultivation fields over old-growth forest between 1987 and 2014. a) Total area cultivated per year, i.e. total area where a clear cut occurred regardless if the previous land cover was an old-growth forest or a secondary-forest fallow (in black), and the area where clear cuts occurred in old-growth forests (in grey). b) The percentage of clear cut area in old-growth forests relative to the total clear cut area each year.

An average of 652 ± 275 ha was clear cut for cultivation each year, 36 ± 15% of which (215 ± 80 ha) was clear cut in old-growth forest (Fig 3A). Although the total area cultivated and the area deforested varied a lot among years (Fig 3A), they showed contrasting trends. While the total area cultivated increased with 19.23 ha per year (_adj_Rsq = 0.28, Df = 26, p = 0.002), the annual deforestation decreased with 17.22 ha per year (_adj_Rsq = 0.12, Df = 26, p = 0.020) (Fig 3A). As a result, the percentage of deforested area in relation to the total cultivated area significantly decreased over time (_adj_Rsq = 0.85, Df = 26, p<0.001; Fig 3B). Altogether, these results show that over time there is more area being cultivated per year, and it is increasingly being done on fallows and not on old-growth forests.

### Determinants of land-use intensity

The age of secondary-forest fallows and the number of clear-cut events were significantly related to the distance to residences and to communities’ size (Table 1). The age of secondary-forest fallows increased with distance to residences and was significantly higher around isolated households than around villages of different sizes (Table 1). A significant interaction term showed that the size-effect of distance to residences varied with communities’ size. The larger the community, the lower was the size-effect (slope) of distance on the age of secondary-forest fallows (Table 1), i.e. the larger the village the more similar was the age of fallows across the landscape. The number of clear-cut events decreased with distance to residences for all community sizes, and it was higher in medium- and large-size villages than in small villages and isolated households (Table 1). There was no significant interaction, showing a similar trend of higher intensity of use closer to the residences for all community sizes.

**Table 1:** Results of the mixed-effects model for age of secondary forests and number of past clear-cut events. In both models, communities are included as random effects. The values of the slope (β) and its standard error (SE), the degrees of freedom (DF), t-value and P-value for each variable are provided. For the dummy variable community size, isolated households are taken by the model as the reference category.

Dependent variable	Independent variables	β	SE	DF	*t-value*	*P-value*
*Age of secondary-forest fallows*						
	(Intercept)	8.62	0.77	2417	11.16	**0.000**
	Distance to residences	0.95	0.32	2417	2.93	**0.003**
	Large village	-1.49	1.16	26	-1.28	0.213
	Medium village	1.38	1.15	26	1.20	0.240
	Small village	-1.20	0.96	26	-1.25	0.224
	Distance*Large village	-0.88	0.37	2417	-2.35	**0.019**
	Distance*Medium village	-2.33	0.50	2417	-4.66	**0.000**
	Distance*Small village	-0.60	0.37	2417	-1.61	0.108
*Number of clear-cut events*						
	(Intercept)	2.25	0.11	2420	19.98	**0.000**
	Distance to residences	-0.15	0.04	2420	-3.44	**0.001**
	Large village	0.47	0.13	26	3.73	**0.001**
	Medium village	0.29	0.12	26	2.34	**0.027**
	Small village	0.17	0.11	26	1.50	0.145

### Changes in fallow-period length over time

**Fig 4:**
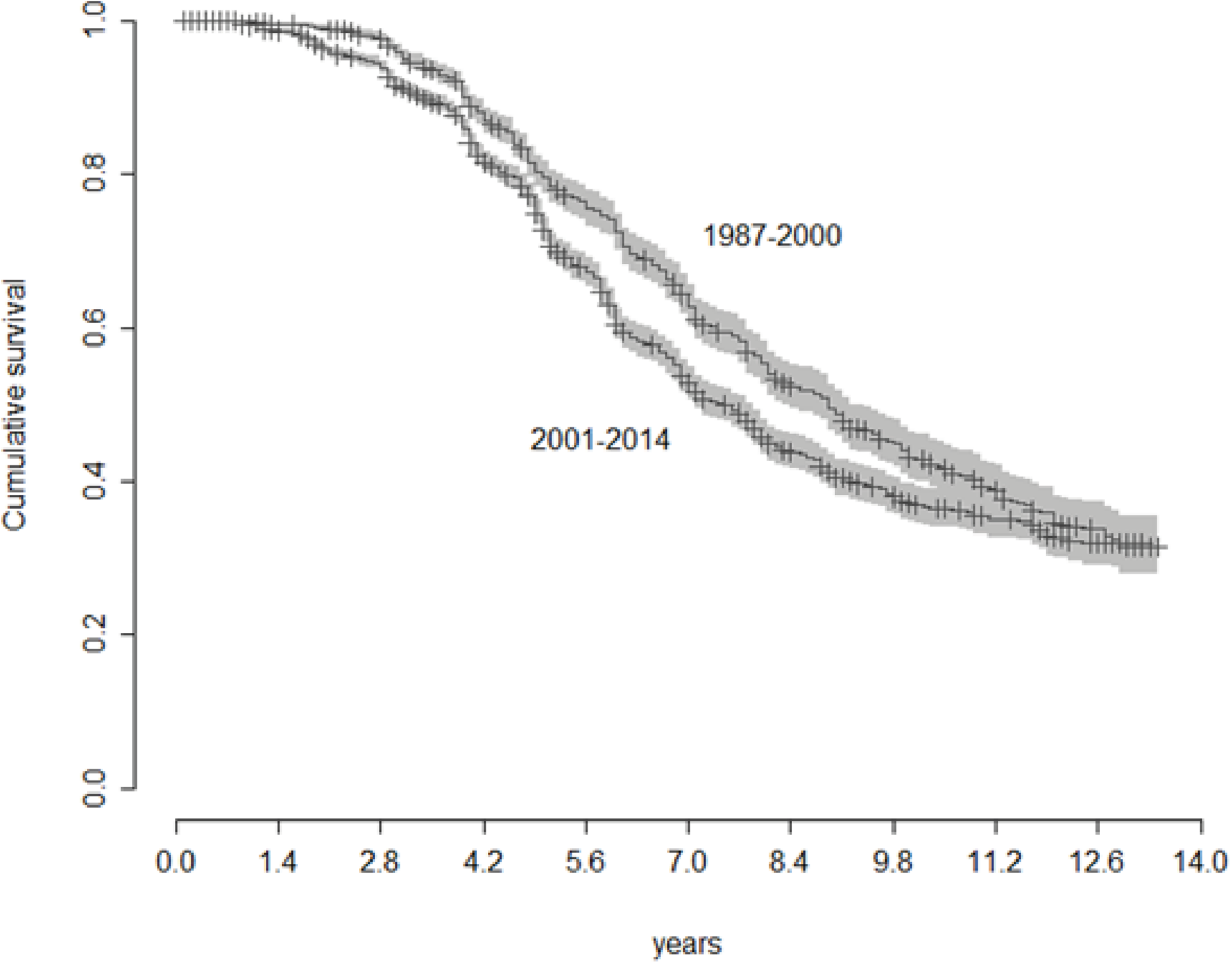
Kaplan-Meier survival curves for fallows fields at two time-periods: 1987–2000 and 2001–2014. Cumulative survival probability of fallows refers to the probability of fallows not been cut, i.e. of continuing regrowth.

The probability of a secondary-forest fallow not been cut (i.e. surviving and continuing to be a fallow) was significantly higher in 1987–2000 than in 2001–2014 (Chi^2^ 1235, Df = 1, P < 0.001; Fig 4). The Kaplan-Meier survival curves show that when time passes (x axis in Fig 4), the probability of survival (i.e. of a fallow continuing to be a fallow) gradually decreases (y axis in Fig 4). The survival curve of fallows in 2001–2014 is lower than in 1987–2000, indicating lower survival probability at a given age. In the past, a secondary-forest fallow 6 yrs old had 75% probability of not been cut whereas after 2001 this probability was reduced to 62% (Fig 4).

**Fig 5:**
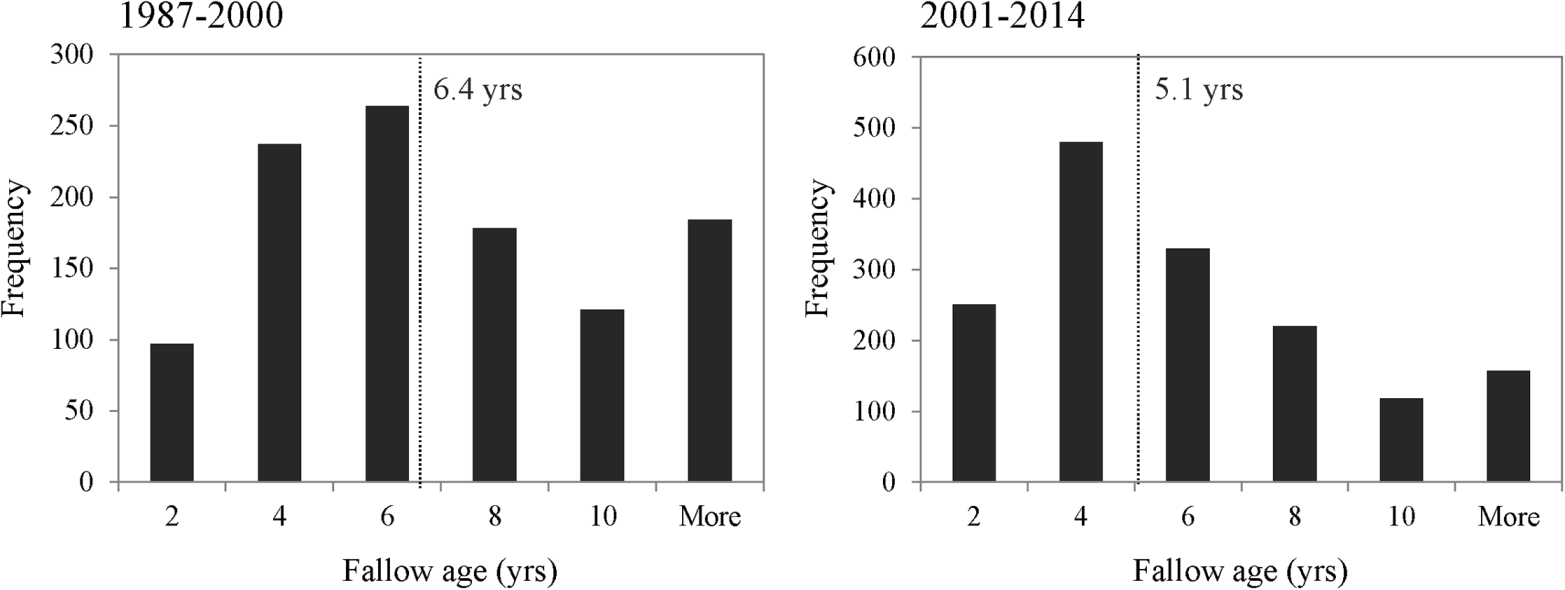
Frequency distribution of fallow ages in two time-periods: 1987–2000 and 2001–2014, in the riverine settlements of the region of the middle-Amazonas river, Brazil. Grey dashed line and the number indicate the average fallow age in each time-period. Note the different scales in y axes of the two graphs.

The average age of fallows decreased over time, from 6.4 ± 4.1 years (MEAN ± SD) in 1987–2000 to 5.1 ± 3.8 years after 2001. Also the frequency distribution of fallow ages in the landscape showed important changes over time (Fig 5). The standard deviation of fallow age decreased, and the frequency of fallows with ≥ 10 years old decreased from 28 to 18% over time (Fig 5).

## Discussion

In this study we assessed the dynamics of shifting cultivation fields over time using a novel remote sensing approach that allowed for high temporal and spatial resolution. Our data show that the expansion of swiddens over old-growth forest has slowed down in the last 30 years, and that swidden cultivation has been intensified, through the shortening of the fallow period. Our data support that the length of the fallow period is associated to access and suggests that constraints of land accessibility are encouraging agricultural intensification. These findings reveal that the dynamics of swidden cultivation is changing in Amazonia, possibly following recent socio-economic transformations in riverine livelihoods related to migration movements and market integration [13, 14].

Our results show that the highest expansion rate of swiddens over old-growth forest occurred in the late 80’s and early 90’s (Fig 3A, Fig 3B), which coincides with the increase in human population in the studied municipalities: 1.2 times increase between 1980 and 1991 vs 0.2 times between 1991 and 2010 (S3 Fig; [27]). Between the 1960’s and 1980’s, migration movements supported by the Brazilian government have brought millions of people from other regions of Brazil to populate Amazonia, leading to high levels of deforestation [38]. After this period, population growth rates decreased and became more dependent on birth and mortality rates and on internal migration movements than on immigration from other regions [13, 38].

After the 90’s the expansion of swiddens over old-growth forest has slowed down, but the area opened for agriculture has increased (Fig 3). The increase in total cultivated area happens, therefore, at the expense of fallows, coinciding with an overall decrease in the length of the fallow period (Fig 4). Considering that there was virtually no law enforcement impeding deforestation in these remote areas during this period, other processes may explain why there was a reduction in deforestation along with an increase in cultivated area and agricultural intensification. A combination of high population densities, limited land accessibility and increased market demand may contribute to explain this pattern.

High-populated villages have higher internal demand for food, more people sharing the space for cultivation and usually these villages are closer to the urban centre and more integrated to the market [13], which altogether motivates intensification [39, 40]. Our results support that idea, showing that more populated villages had higher frequency of cycles and shorter fallow over the entire cultivated landscape than smaller villages (Table 1). In smaller villages, the frequency of cycles is lower and short fallow periods were only practiced at fields close to the residences (Table 1). Such relation between population density and intensification is usually mediated by limited access to new land for cultivation [41]. Although in riverine Amazonia there is plenty of unoccupied and virtually available land, access to it is limited by walking distances and reduced navigability in the dry season [10]. In this context, land scarcity is related to accessibility. Distance to access has been shown to be an important driver of land use type and intensity [40, 42–44].

In riverine Amazonia, access limitation encourages intensification also because the transport of harvested cassava to the processing place (*casa de farinha*), and of *farinha* to the residences, is done manually. Carrying the produce over longer distances [43]) is unpractical and inhabitants of the study area acknowledged that fallow period has been recently shortened because “forest is too far” to open new fields (c.f. [13, 42]). But how far is too far? Most swidden-fallow fields (80%) are accessed by land and are located within 2 km from the residences area (Fig 1; S2 Fig), and most new fields are being opened in old-growth forests upriver along small but navigable tributaries (Fig 1B), confirming the impact of accessibility on land cover change and land-use patterns [9, 43–45].

Along with land accessibility, other factors related to socio-economic changes, policies, regulations and land tenure may also play a role [8, 42, 46, 47]. Major changes in the socio-economy of riverine Amazonia are related to the resettlement of the rural population around villages and urban centres and to the increased market integration of smallholder farmers [13, 14, 48]. Market demand for *farinha*, the regional staple food, has been sharply increasing over the last decade followed by a price increase of 398% against an accumulated inflation of 72.3% over the same period [49]. Increasing market demand and prices have been stimulating cassava cultivation for both commercialization and subsistence, as living costs increase with staple food prices [48]. Aiming to increase yield and to supply demand, rural policies are encouraging the production of cassava and its intensification towards fertilized monocultures [50]. But, in practice, the low income in riverine Amazonia do not allow farmers to acquire fertilizers, hence they end up following a monoculture model but achieving low productivity [15].

Repeated cycles within a short-fallow-period regime results in lower regrowth rate of fallows, lower productivity of swiddens and long lasting soil degradation [15, 18, 51]. Swiddens productivity decreases due to reduced crop yield and increased labour demand for weeding as open-area grasses become a burden [15]. As a result of agricultural intensification, young secondary-forest fallows predominate (80% is less than 12 years old and 50% is younger than 5 years old; Fig 2) and older patches disappear from the landscape. Ephemeral secondary forests play a restricted role in the conservation of tree species [52], carbon sequestration [53, 54] and the provision of forest products [55]. Therefore, intensification undermines the potential of these landscapes to provide ecosystem services such as carbon sequestration, biodiversity conservation and agricultural production, jeopardizing the sustainability of shifting cultivation landscapes. This study shows that intensification and expansion are processes occurring simultaneously in the landscape (*c*.*f*. [7]), but with its relative importance changing over time. Nowadays, intensification is the prevailing process in the landscape. Land accessibility plays an important role here, constraining the areal expansion of swiddens and encouraging local intensification together with other drivers such as concentration of human population in villages closer to urban centres [13], low accessibility [10], increasing market demand for *farinha* and policy incentives for intensification [50]. Such drivers are present all over riverine Amazonia, and therefore agricultural intensification is likely a reality in the many cassava-producing villages. Land-use planning that take into account the constraints and opportunities of riverine agriculture is, therefore, increasingly needed to avoid the negative consequences of the ongoing agricultural intensification for livelihoods and the environment in riverine Amazonia.

## Supporting information

S1 Fig Example of a temporal profile spanning from 1984 to 2015, and parameters used for land-use change detection. Temporal profile is built from the satellite image observations (green dots) over time. Abrupt changes in NDMI are identified as breakpoints (red-dashed lines), which separates two segments (blue lines represent the best linear model fitted for each segment). Segments are characterized by their duration (yellow solid line), magnitude (red solid line) and slope (β). The two classes of breakpoints are indicated: clear-cut break (1 and 3) and stabilization break (2 and 4).
(TIF)

S2 Fig Distribution of swidden-fallow fields in relation to distance to residences. The cumulative number of samples located at different distances to the nearest of community are plotted. The 31 communities are represented by different colours. Black dashed lines indicate average and standard deviation of the distance containing 80% of the fields (2.06 ± 1.65 km; Mean ± SD).
(TIF)

S3 Fig Rural and urban population of the region of the middle-Amazon river, Brazil. Rural and urban population of the three municipalities (Tefé, Alvarães and Uarini) estimated by the national census of 1980, 1991, 2000 and 2010 (IBGE, 2013). In 1980, the three municipalities belonged to the municipality of Tefé. Therefore, for comparison we show the estimated population of the three municipalities together, in the four censuses.
(TIF)
