# Supplementary figures and images for: Spatial and temporal dynamics of shifting cultivation in the middle-Amazonas river: Expansion and intensification
Source: PLoS One. 2017 Jul 20;12(7):e0181092. doi: 10.1371/journal.pone.0181092 (PMC5519060; doi:10.1371/journal.pone.0181092)

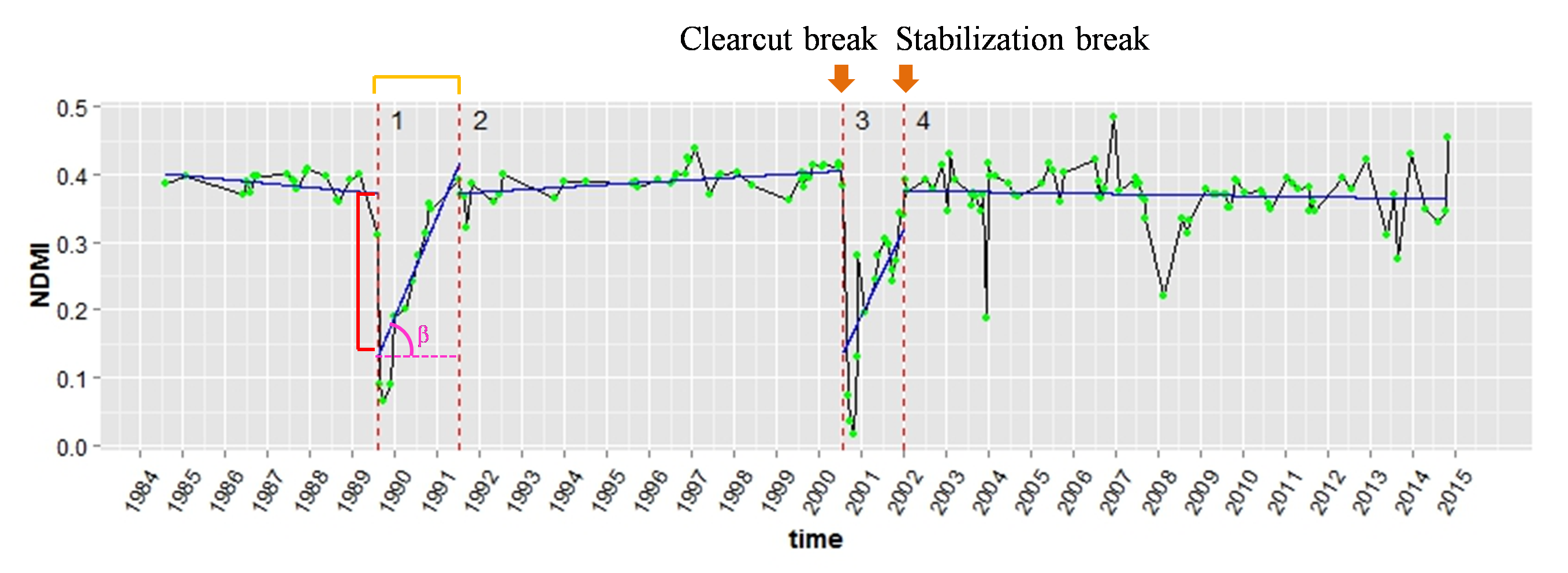

Supplement: S1 Fig — Temporal profile is built from the satellite image observations (green dots) over time. Abrupt changes in NDMI are identified as breakpoints (red-dashed lines), which separates two segments (blue lines represent the best linear model fitted for each segment). Segments are characterized by their duration (yellow solid line), magnitude (red solid line) and slope (β). The two classes of breakpoints are indicated: clear-cut break (1 and 3) and stabilization break (2 and 4). (TIF) [file pone.0181092.s001.tif]

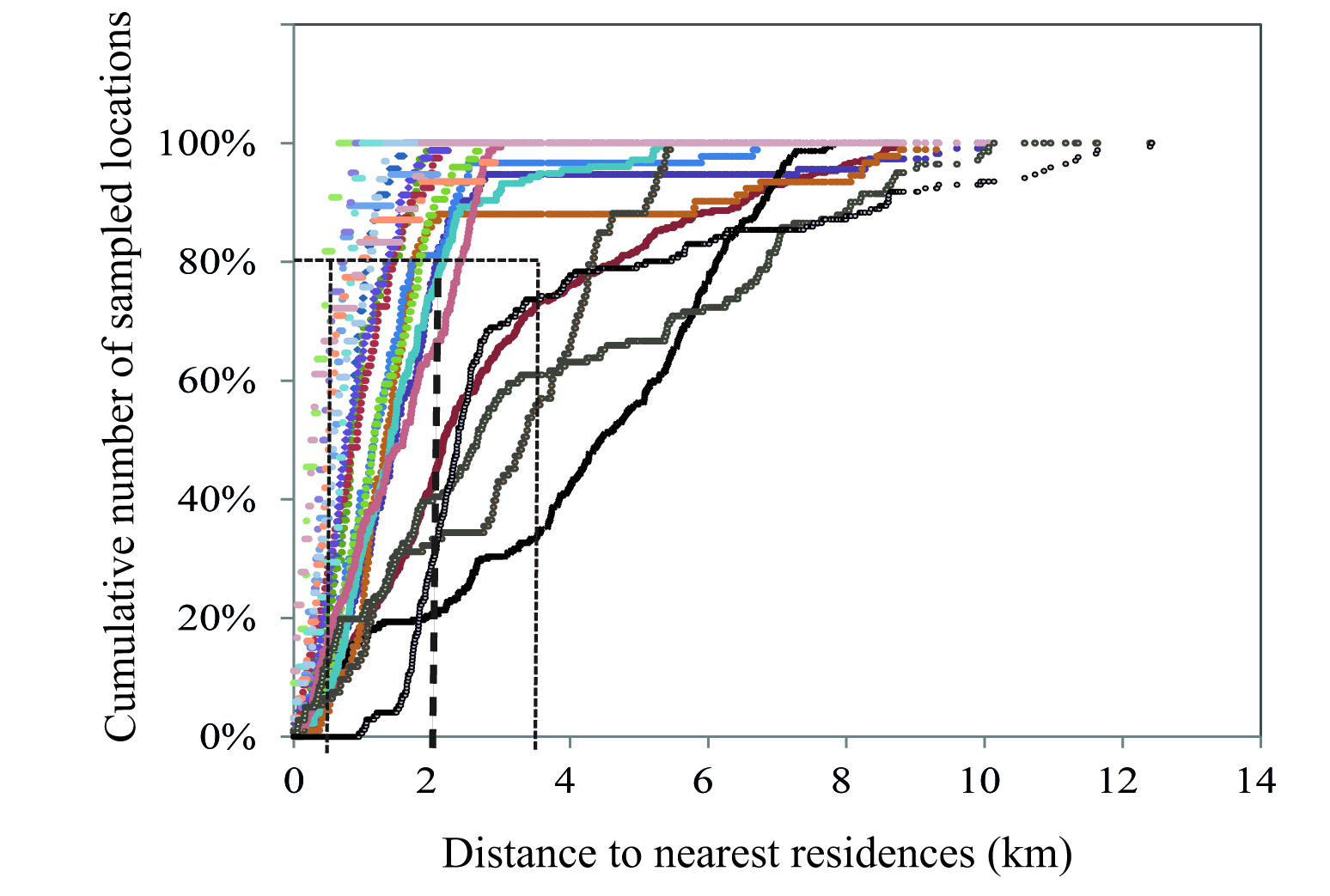

Supplement: S2 Fig — The cumulative number of samples located at different distances to the nearest of community are plotted. The 31 communities are represented by different colours. Black dashed lines indicate average and standard deviation of the distance containing 80% of the fields (2.06 ± 1.65 km; Mean ± SD). (TIF) [file pone.0181092.s002.tif]

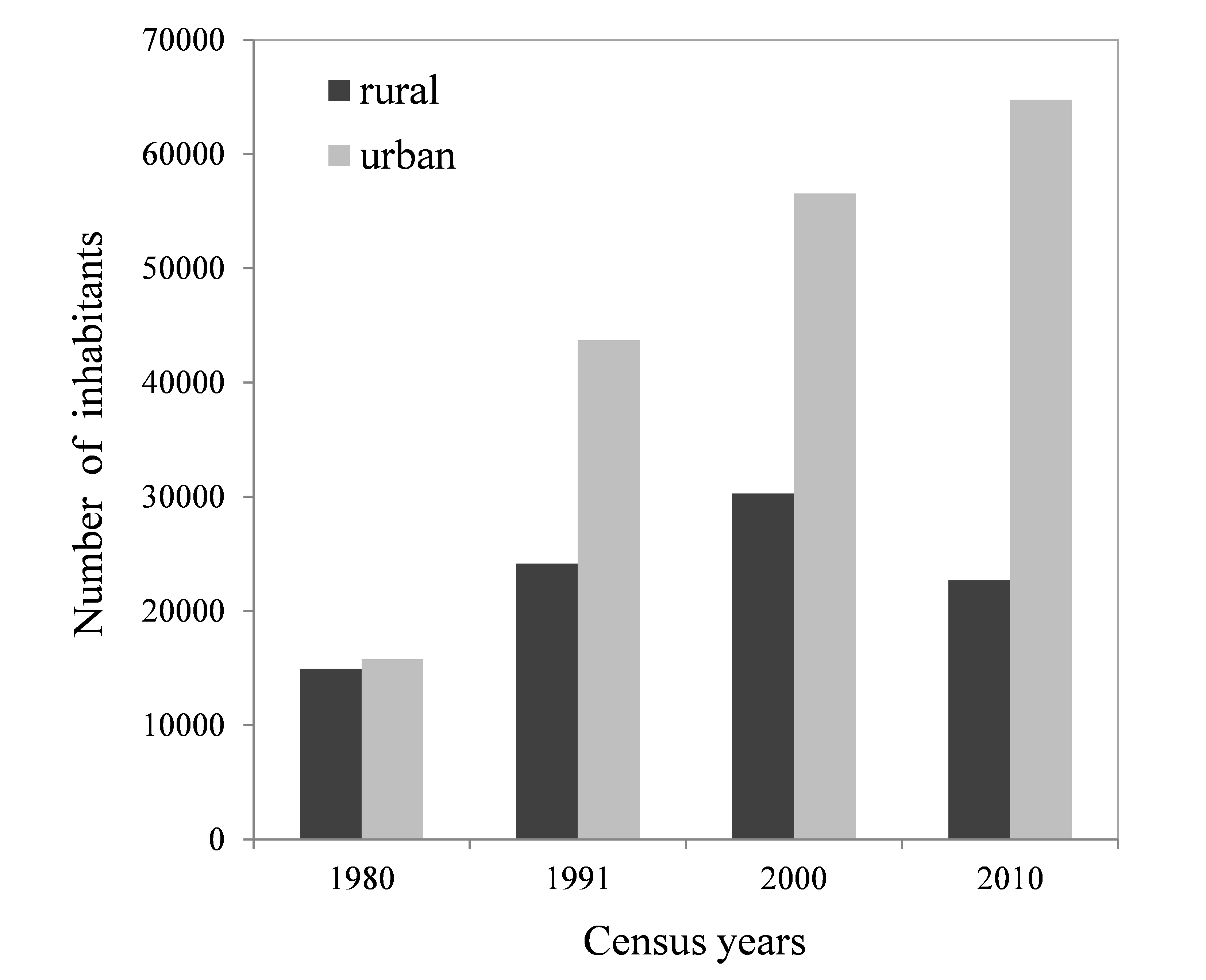

Supplement: S3 Fig — Rural and urban population of the three municipalities (Tefé, Alvarães and Uarini) estimated by the national census of 1980, 1991, 2000 and 2010 (IBGE, 2013). In 1980, the three municipalities belonged to the municipality of Tefé. Therefore, for comparison we show the estimated population of the three municipalities together, in the four censuses. (TIF) [file pone.0181092.s003.tif]
